# Mapping the neurobiological landscape linking obesity with selected psychiatric conditions: global trends and emerging hotspots

**DOI:** 10.1080/16549716.2026.2749297

**Published:** 2026-10-29

**Authors:** Chang Liu, Chaonan Dong, Xiang Zhou, Qi Shu, Fan Zhang, Ronglin Cai, Qing Yu

**Affiliations:** a The First Clinical Medical College, Anhui University of Chinese Medicine, Hefei, China; b College of Acupuncture-Moxibustion and Tuina, Anhui University of Chinese Medicine, Hefei, China; c College of Traditional Chinese Medicine, Anhui University of Chinese Medicine, Hefei, China; d School of Integrated Chinese and Western Medicine, Anhui University of Chinese Medicine, Hefei, China; e Key Laboratory of Xin’an Medicine, Ministry of Education, Anhui University of Chinese Medicine, Hefei, China; f Anhui Province Key Laboratory of Meridian Viscera Correlationship, Anhui University of Chinese Medicine, Hefei, China; g Center for Xin’an Medicine and Modernization of Traditional Chinese Medicine, Institute of Health and Medicine, Hefei Comprehensive National Science Center, Hefei, China

**Keywords:** Bibliometrics, neuroinflammation, gut-brain axis, hypothalamic-pituitary-adrenal (HPA) axis, nutritional psychiatry

## Abstract

**Background:**

Obesity frequently co-occurs with a range of psychiatric conditions, creating an important global public health challenge. However, neurobiological research on these associations remains fragmented across disciplines, and a systematic bibliometric synthesis is lacking.

**Objective:**

To map the knowledge structure, identify research hotspots, and characterize temporal trends in neurobiological research linking obesity with selected psychiatric conditions.

**Methods:**

Relevant literature (2010–2025) was retrieved from the Web of Science Core Collection. Following screening, 476 studies were included. Bibliometric and visualization analyses were conducted using VOSviewer, CiteSpace, and Scimago Graphica. A supplementary Scopus analysis assessed publication overlap and concordance in selected bibliometric indicators.

**Results:**

Publications surged after 2021, with the last 5 years accounting for 54.2% of the total. The United States and China had the highest publication counts. Among the 10 most productive countries, France had the highest mean annualized citations per publication. Core hotspots include neuroinflammation, the gut-brain axis, hypothalamic-pituitary-adrenal (HPA) axis dysfunction, and high-fat diet.

**Conclusion:**

Neuroinflammation, the gut–brain axis, and HPA-axis-related processes emerged as recurring themes. These findings highlight the need for further investigation of metabolic, immune, and neural processes in geographically diverse populations, particularly in developing regions.

## Paper Context

**Main findings**: Research on the neurobiological links between obesity and selected psychiatric conditions has grown markedly since 2021, with neuroinflammation and the gut–brain axis emerging as prominent research themes.

**Added knowledge**: This bibliometric analysis maps the geographic, collaborative, and thematic structure of this interdisciplinary field over 15 years and identifies major areas of research concentration and emerging themes.

**Global health impact for policy and action**: The findings highlight the need for more geographically diverse and context-sensitive research before neurobiological insights are translated into population-level strategies addressing obesity-associated psychiatric conditions.

## Background

Obesity and mental disorders have emerged as two core challenges facing global public health in the 21st century [1,2]. Both exhibit rapidly increasing prevalence and high clinical comorbidity, forming a complex bidirectional relationship that poses unique difficulties for disease prevention and control [3]. Global obesity prevalence rose from 6.11% (95% uncertainty interval [UI], 5.42%–6.87%) in 1990 to 16.06% (14.26%–17.99%) in 2022 and is projected to reach 38.96% (37.85%–40.08%) by 2040. More than 70% of people with obesity now live in developing countries, and the burden is shifting toward lower-income settings [4]. The global burden of anxiety and depressive disorders has also increased substantially [5].

Obesity and psychiatric disorders frequently coexist in clinical populations. Large-scale epidemiological studies have documented associations between severe obesity and several psychiatric conditions, including depression, anxiety, bipolar disorder, and schizophrenia [6]. Conversely, metabolic abnormalities and obesity are also common among individuals with severe psychiatric illness [7,8]. Such comorbidity can complicate treatment and long-term management, as illustrated by a recent report of bariatric surgery in a patient with class III obesity and bipolar disorder [9]. Importantly, such clinical observations demonstrate comorbidity and management complexity but do not establish the neurobiological mechanisms underlying these associations.

Separately from this clinical evidence, a growing body of experimental and mechanistic research has investigated biological processes that may contribute to the association between obesity and psychiatric conditions. Proposed pathways include chronic low-grade inflammation, hypothalamic–pituitary–adrenal axis dysregulation, insulin and leptin resistance, and altered gut microbiota–brain communication [10–15]. These mechanisms may participate in both metabolic regulation and affective or behavioral processes, providing potential biological frameworks for understanding the observed comorbidity. However, evidence across these pathways remains distributed among multiple disciplines, and their relative contributions and causal relationships have not been fully established.

Bibliometric analysis can quantify publication patterns, identify influential contributors and literature, and track thematic evolution [16–18]. It is therefore well suited to mapping this fragmented interdisciplinary field. We conducted a bibliometric analysis of research on the neurobiological links between obesity and selected psychiatric conditions. We mapped publication trends, leading countries, institutions and authors, highly cited literature, thematic clusters, and emerging frontiers to characterize the field’s knowledge structure and inform future research.

Previous bibliometric studies have addressed adjacent topics, including obesity–depression comorbidity [19], obesity and psychology [20], and the gut–brain axis in depression [21], but have generally focused on a single disorder, pathway, or specific population. The present study therefore provides a broader bibliometric overview of neurobiological research linking obesity with selected psychiatric conditions.

## Methods

### Data sources and search strategy

On 3 January 2026, we conducted a search in the Science Citation Index Expanded (SCI-EXPANDED) of the Web of Science Core Collection (WoSCC) for publications examining neurobiological links between obesity and selected psychiatric conditions. The search covered the period from 1 January 2010 to 31 December 2025. The following search strategy was applied: TS=((obes* OR overweight OR ‘body mass index’ OR BMI) AND (‘mental disorder’ OR ‘psychiatric disorder’ OR ‘psychological distress’ OR anxi* OR depress* OR ‘mood disorder’ OR ‘bipolar disorder’ OR ‘stress disorder’ OR PTSD OR ‘post-traumatic stress disorder’ OR ‘chronic stress’) AND (‘neurobiolog’ OR neurocircuit OR neuroinflamm* OR ‘HPA axis’ OR ‘hypothalamic-pituitary-adrenal’ OR ‘gut-brain axis’ OR microbiota OR ‘neural pathway*’)) (full details in Supplementary Table 1). The psychiatric search block combined broad umbrella terms with condition-specific terms to balance search breadth and sensitivity. Broad terms such as ‘mental disorder’ and ‘psychiatric disorder’ allowed relevant studies involving a wider range of psychiatric conditions to be retrieved, whereas the condition-specific terms primarily targeted depression, anxiety, mood disorders, bipolar disorder, and stress-related conditions. Accordingly, the term ‘selected psychiatric conditions’ is used throughout this manuscript to refer to the heterogeneous but non-exhaustive set of psychiatric disorders, symptoms, and related phenotypes captured by the predefined search strategy and retained after relevance screening.

The initial search yielded 1,745 records. After restricting the results to English-language publications and removing retracted publications, conference abstracts, Early Access records, and other ineligible document types, 1,677 records remained. These 1,677 records were subsequently subjected to manual eligibility assessment. Early Access records were excluded to maintain consistency in bibliographic metadata and publication-year assignment across the dataset, as these records may not yet have been assigned finalized volume, issue, or pagination information at the time of retrieval.

### Study selection and eligibility assessment

Subsequently, two investigators independently screened the titles and abstracts of the 1,677 remaining records using predefined eligibility criteria. Studies were eligible if they substantively addressed obesity, overweight, or an obesity-related metabolic phenotype; examined one or more psychiatric conditions, symptoms, or related phenotypes captured by the predefined search strategy; and investigated a neurobiological link between the metabolic and psychiatric domains. Studies were excluded if they met any of the following mutually exclusive primary criteria: (1) core topic mismatch; (2) absence of a substantive neurobiological link between obesity and psychiatric conditions captured by the predefined search strategy; or (3) peripheral mention or insufficient relevance, defined as cases in which the obesity–psychiatric relationship was mentioned only incidentally and was not substantively investigated in the study objectives, analyses, results, or mechanistic interpretation. Records that could not be reliably classified at this stage underwent full-text assessment using the same criteria. After excluding 1,082 records at the title/abstract stage, 595 records underwent full-text assessment, of which 119 were further excluded, leaving 476 publications for the final analysis. Inter-reviewer agreement before consensus was 90.3% (Cohen’s κ = 0.79) at the title/abstract stage and 94.8% (κ = 0.84) at the full-text stage. Disagreements were resolved through discussion, with unresolved cases adjudicated by a third investigator. Further details of the screening process are provided in Supplementary Table 2.

### Data standardization

Before analysis, country/region labels and keywords were standardized using a prespecified thesaurus. For the primary country-level analysis, the unit of analysis was the sovereign state; accordingly, England, Scotland, Wales, and Northern Ireland were mapped to the United Kingdom. People’s R China and Taiwan were consolidated under China in the primary aggregated analysis. When a publication contained affiliations from more than one label mapped to the same entity, it was counted only once for that entity. Publication counts, citation counts, and network indicators were recalculated after aggregation rather than obtained by directly summing the corresponding values. Keyword normalization covered capitalization, hyphenation, singular and plural forms, spelling variants, and abbreviations. Conceptually related but non-equivalent terms were retained separately. The complete country/region and keyword mappings are provided in Supplementary Table 3. To assess the influence of geographical aggregation, country/region-level indicators were recalculated without aggregation using otherwise identical analytical settings (Supplementary Table 4). Author and institutional labels were retained as imported into VOSviewer, without additional manual merging. This conservative approach was adopted to reduce the risk of conflating different individuals or institutions on the basis of similar names alone. For institutional‑level analysis, each distinct institution listed in the affiliations of a publication was assigned one publication credit; that is, one paper with multiple institutional affiliations would contribute to the publication count of every affiliated institution. No fractional counting was applied for institutional publication or citation metrics.

### Selection and application strategy of visualization tools

We used VOSviewer (version 1.6.20), CiteSpace (advanced version 6.4.R1), and Scimago Graphica (version 1.0.53) for complementary network and visualization analyses [22–24]. VOSviewer generated collaboration, citation, co-citation, and keyword co-occurrence networks using full counting and association strength normalization. Clusters were identified using the VOS clustering technique, with a resolution of 1.00 and a minimum cluster size of 1 node. Network-specific inclusion thresholds are reported in the figure legends and were selected to retain recurrent contributors or frequently occurring items while reducing visual clutter and maintaining interpretability. Scimago Graphica supported geographic and network presentation. CiteSpace was used for data preprocessing. Node size represented activity, and line thickness represented link strength. CiteSpace generated subject-category and reference clusters, keyword timelines, and burst analyses of categories, keywords, and references. CiteSpace analyses covered 2010–2025 using 1-year time slices and g-index node selection with k = 25. Additional selection settings were LRF = 2.5, L/N = 10, LBY = 5, and e = 1.0. No pruning was applied to the WoSCC networks. Burst detection used two states, with α_1_/α_0_ = 2.0, α_i_/α_i-1_ = 2.0, and a minimum burst duration of 2 years. For exploratory visualization, γ was adjusted separately for each burst analysis to obtain an analysis-specific target number of burst items. This output-count-based choice served presentation purposes rather than identifying an optimal detection threshold. Network-specific modularity Q and weighted mean silhouette S values are reported in the corresponding figures. Microsoft Excel (Office 2021) was used to manage and standardize records and prepare tables. Figure 1 summarizes screening and analysis.

**Figure 1.**
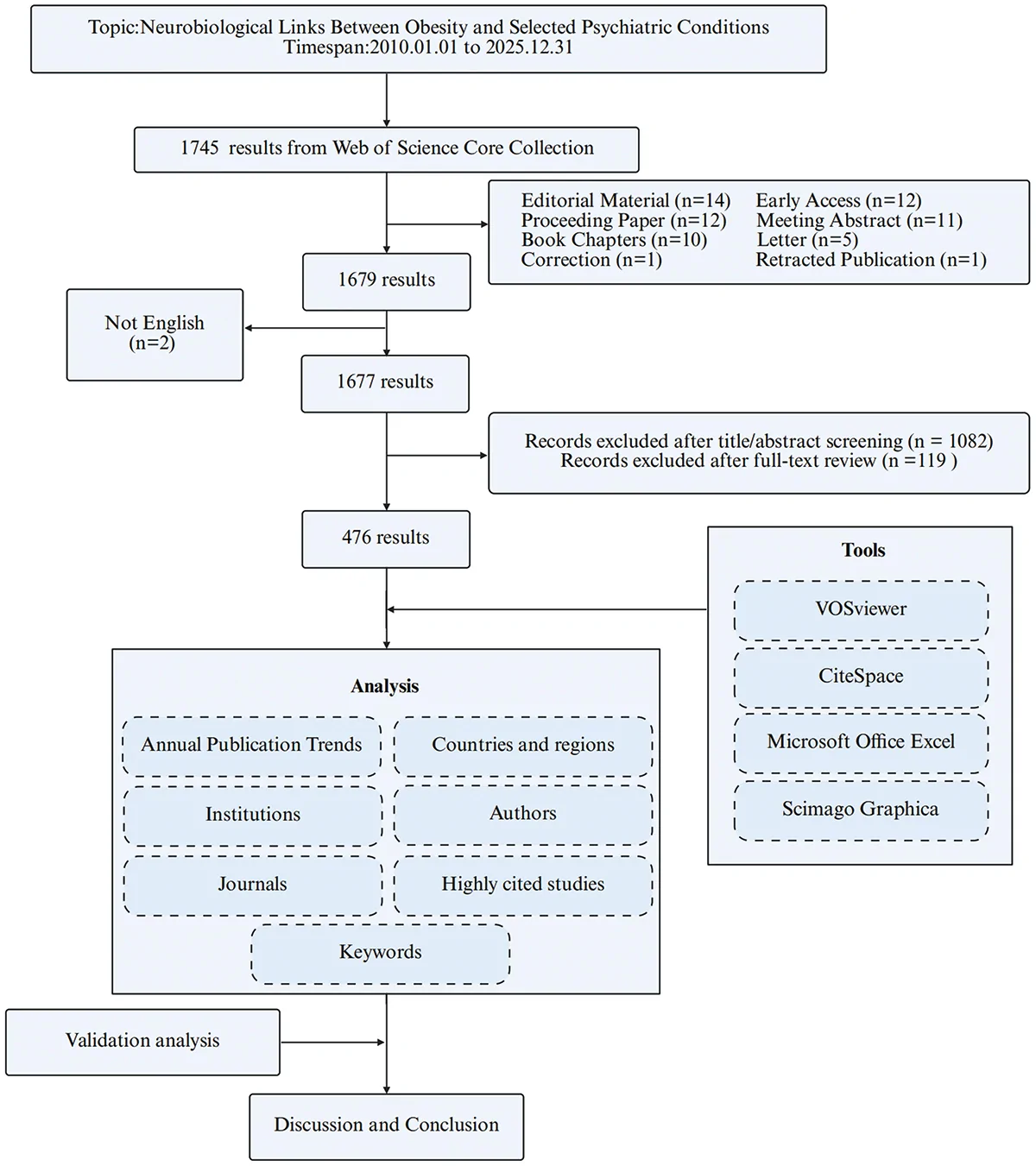
Flowchart of the paper screening and analysis process.

### Cross-database robustness assessment

A supplementary Scopus search was conducted on 12 June 2026, using a database-adapted strategy targeting the same conceptual domains as the WoSCC search. Retrieval covered English-language publications from 1 January 2010, to 31 December 2025. The complete Scopus query and screening restrictions are provided in Supplementary Table 1. The searches were conceptually aligned rather than technically identical. Publication overlap was determined by normalized DOI matching, supplemented by title, author, and publication-metadata checks when DOI information was unavailable. Spearman’s rank correlation, with average ranks for ties, was used to assess concordance in annual publication counts and publication counts across the 10 countries selected for comparison. The Jaccard similarity coefficient, defined as the intersection divided by the union, quantified overlap between the publication sets and between the selected high-frequency keyword sets. These comparisons provided a partial robustness assessment of selected bibliometric patterns rather than an independent validation of all findings.

## Results

### Publication trends and distribution

The final selection of 476 documents consisted of 330 articles (69.33%) and 146 reviews (30.67%). Figure 2 illustrates the growth trajectory of publications related to the neurobiological mechanisms of obesity and selected psychiatric conditions, indicating a significant increase in research interest in this field. Annual output remained relatively stable from 2010 to 2020 and then increased sharply after 2021; the most recent five-year period (2021–2025) contributed 54.2% of the final corpus.

**Figure 2.**
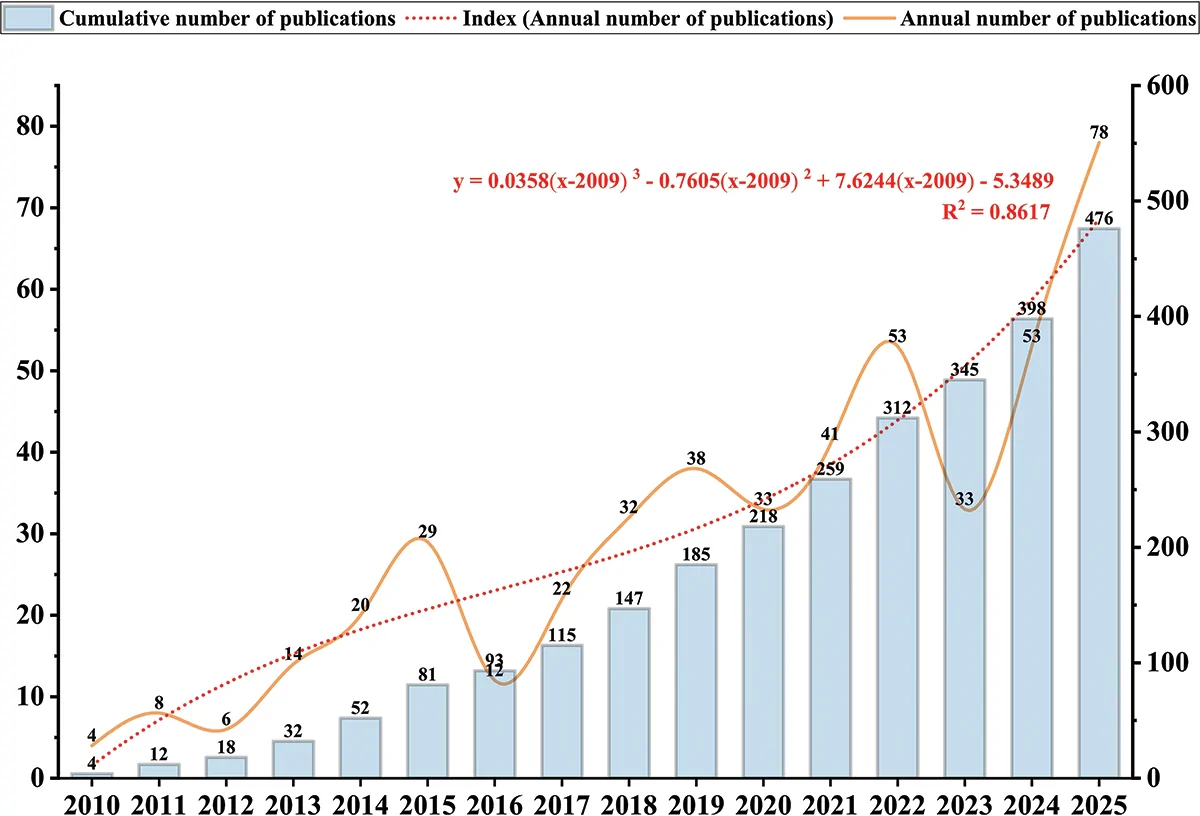
Line chart of annual publication volume (showing the growth trend).

### Country and institution analysis

A total of 63 countries contributed publications on the neurobiological mechanisms linking obesity with selected psychiatric conditions. The United States and China led publication output, with 120 and 86 publications, respectively (Table 1). The United States had the highest total citation count (9,885). Among the 10 most productive countries, France had the highest mean annualized citations per publication (17.41), followed by Australia (16.68) and Canada (14.75); the corresponding values for the United States and China were 11.89 and 8.39. Rankings by publication count were broadly consistent with and without geographical aggregation: the United States and China remained first and second, respectively, while England ranked eighth in the unaggregated analysis, corresponding to the position of the United Kingdom in the aggregated analysis (Supplementary Table 4). Figure 3 presents the geographical distribution of publications and the country-level collaboration and citation networks. Although international collaboration was evident, it remained concentrated among a small group of countries.

**Figure 3.**
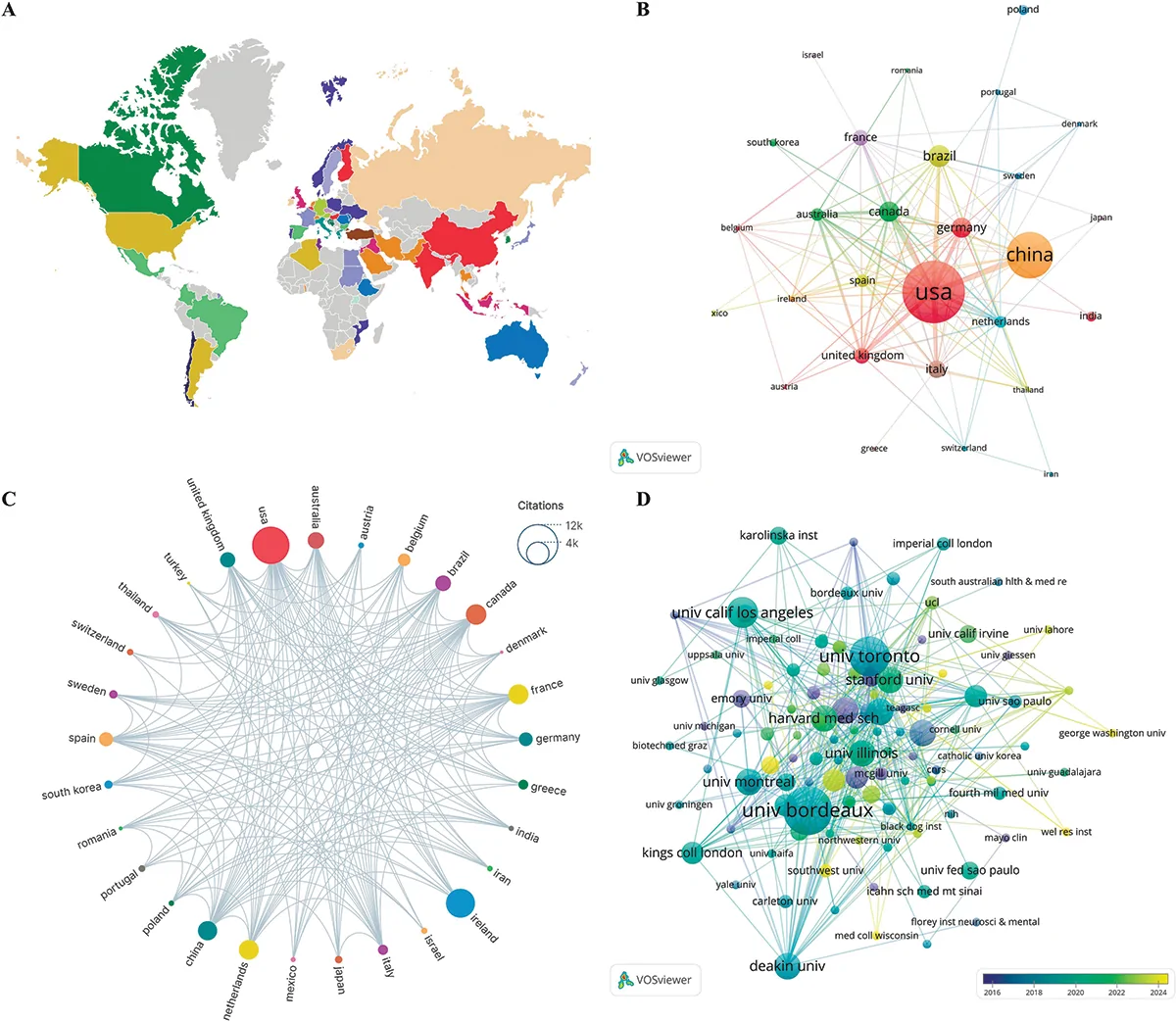
Visualization of countries and institutions involved in research on the neurobiological mechanisms of obesity and selected psychiatric conditions. A: Global distribution of research findings; B: Collaboration network of countries with at least 5 publications; C: Citation network of countries with at least 5 publications; D: Collaboration network of institutions with at least 2 publications.

**Table 1.** Top 10 most productive countries in terms of publication volume.

Rank	Countries	Publications	Citations	Average citations	Mean annualized citations per publication	Total link strength
1	United States	120	9885	82.38	11.89	76
2	China	86	2578	29.98	8.39	26
3	Brazil	38	1728	45.47	9.81	30
4	Canada	34	2954	86.88	14.75	50
5	Germany	34	1314	38.65	5.40	33
6	Italy	29	724	24.97	6.04	32
7	France	26	2778	106.85	17.41	24
8	United Kingdom	26	1608	61.85	12.92	43
9	Australia	20	1928	96.40	16.68	36
10	Spain	20	1385	69.25	13.30	38

According to the VOSviewer analysis, the 476 included publications involved 912 institutions, with the top 10 contributing institutions summarized in Table 2. These leading institutions were mainly located in France, Canada, the United States, and China, collectively contributing 70 publications. Regarding citation performance, the three institutions with the highest total citation counts were Chengdu University of Traditional Chinese Medicine (1,095 citations), Stanford University (1,059 citations), and the University of Melbourne (854 citations). Stanford University exhibited the highest average citation rate among the top institutions, with 176.50 citations per paper. Figure 3(D) presents the institutional collaboration network, showing extensive connections among institutions investigating the neurobiological mechanisms of obesity and selected psychiatric conditions. The University of Bordeaux and the University of Toronto displayed multiple collaboration links within the institutional network. In addition, relatively dense connections were also visible among several institutions in the yellow and green clusters.

**Table 2.** Top 10 most productive institutions in terms of publication volume.

Rank	Institutions	Publications	Citations	Average citations	Countries
1	University of Bordeaux	11	768	69.82	France
2	University of Toronto	9	767	85.22	Canada
3	Chengdu University of Traditional Chinese Medicine	7	1095	156.43	China
4	University of California, Los Angeles	7	626	89.43	United States
5	Stanford University	6	1059	176.50	United States
6	University of Melbourne	6	854	142.33	Australia
7	University of Cincinnati	6	599	99.83	United States
8	University of Montreal	6	297	49.50	Canada
9	Institut National de la Recherche Agronomique	6	149	24.83	France
10	University of Illinois	6	119	19.83	United States

### Author analysis

VOSviewer identified 3,006 authors contributing to publications in this field, of whom 63 published at least three papers. Table 3 lists the top 10 authors ranked by publication output. Lucile Capuron and Nathalie Castanon, both from the University of Bordeaux, jointly led publication output with nine publications each. Roger S. McIntyre from the University of Toronto followed with eight publications. Among the authors listed in Table 3, Lucile Capuron had the highest total citations and mean citations per publication. VOSviewer was used to construct collaboration networks among core authors and co-cited authors in the field of neurobiological mechanisms of obesity and selected psychiatric conditions (Figure 4). Figure 4(A) presents the collaboration network of 63 authors with at least three publications. Several author clusters were identified, although connections between clusters were relatively limited. Figure 4(B) illustrates the author co-citation network, which comprised three major clusters represented by different colors. Of the 26,325 co-cited authors identified, 68 met the inclusion threshold of at least 30 citations. Within this dataset, Michael Maes was the most frequently co-cited author, with a co-citation count of 96.

**Figure 4.**
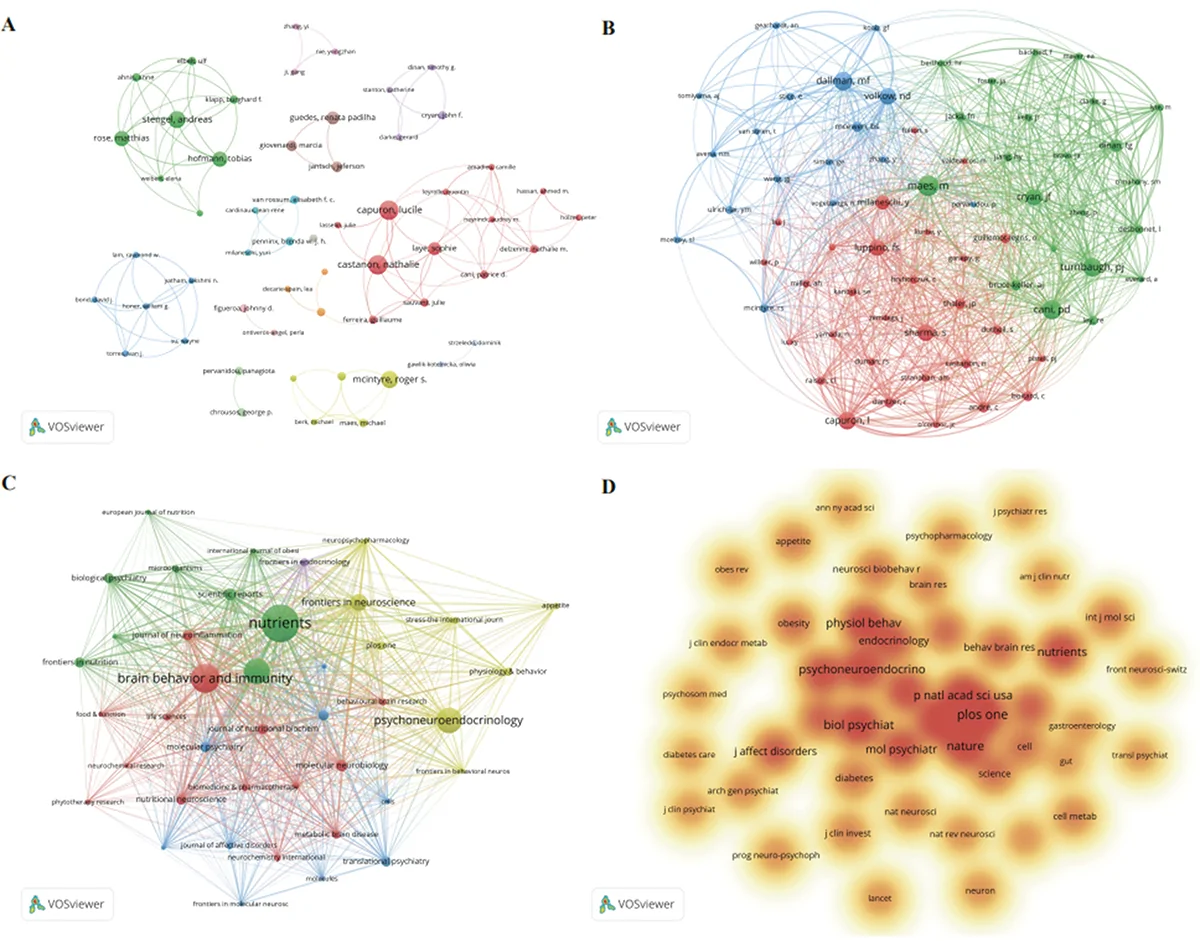
Bibliometric network visualizations of core authors and journals in research on the neurobiological mechanisms underlying obesity and selected psychiatric conditions. A: Visualization of authors with at least 3 publications; B: Co-citation network of authors with at least 30 citations; C: Visualization of journals with at least 3 publications; D: Visualization of co-cited journals with at least 200 citations.

**Table 3.** Top 10 authors by publication output.

Rank	Authors	Publications	Citations	Average citations	Organizations
1	Lucile Capuron	9	1506	167.33	University of Bordeaux
2	Nathalie Castanon	9	828	92.00	University of Bordeaux
3	Roger S McIntyre	8	694	86.75	University of Toronto
4	Andreas Stengel	8	216	27.00	Charité – University Medicine Berlin
5	Tobias Hofmann	7	196	28.00	Charité – University Medicine Berlin
6	Matthias Rose	7	151	21.57	Charité – University Medicine Berlin
7	Sophie Laye	6	524	87.33	University of Bordeaux
8	Renata Padilha Guedes	6	63	10.50	Federal University of Health Sciences of Porto Alegre
9	Jeferson Jantsch	5	53	10.60	Federal University of Health Sciences of Porto Alegre
10	Marcia Giovenardi	5	52	10.40	Federal University of Health Sciences of Porto Alegre

### Journal analysis

VOSviewer analysis identified 245 journals that published studies related to the neurobiological mechanisms of obesity and selected psychiatric conditions, of which 41 journals met the threshold of at least three publications. Figure 4(C) presents the journal citation network, comprising five major clusters represented by different colors. The top 10 journals ranked by publication output are summarized in Supplementary Table 5. Together, these journals contributed 111 articles, accounting for 23.32% of the total publications. Nutrients (22 publications), Brain, Behavior, and Immunity (17 publications), International Journal of Molecular Sciences (15 publications), and Psychoneuroendocrinology (15 publications) were the leading journals by publication volume. Based on citation analysis, Nutrients received 620 citations across 22 publications. Biological Psychiatry showed the highest average citation rate among the analyzed journals, with 113.67 citations per paper. Among the 4,691 cited journals, 47 received more than 200 citations. Supplementary Table 6 lists the most frequently cited journals, with PLoS One (789 citations), Brain, Behavior, and Immunity (760 citations), and Biological Psychiatry (682 citations) ranking highest. Figure 4(D) displays the density visualization of journals with at least 200 citations, where darker colors represent higher citation density.

### Thematic classification analysis

CiteSpace was used to perform burst detection analysis on the 476 included publications to identify rapidly emerging research topics in the field of neurobiological mechanisms underlying obesity and selected psychiatric conditions. Figure 5 illustrates the top 10 subject categories with the strongest citation bursts. The red timeline represents the duration of each burst, whereas burst strength reflects the intensity of citation activity associated with the corresponding category. Among these categories, ‘ENDOCRINOLOGY & METABOLISM’, ‘PSYCHIATRY’, and ‘BEHAVIORAL SCIENCES’ exhibited the highest burst strengths, indicating that these disciplines received substantial attention during the development of this research field.

**Figure 5.**
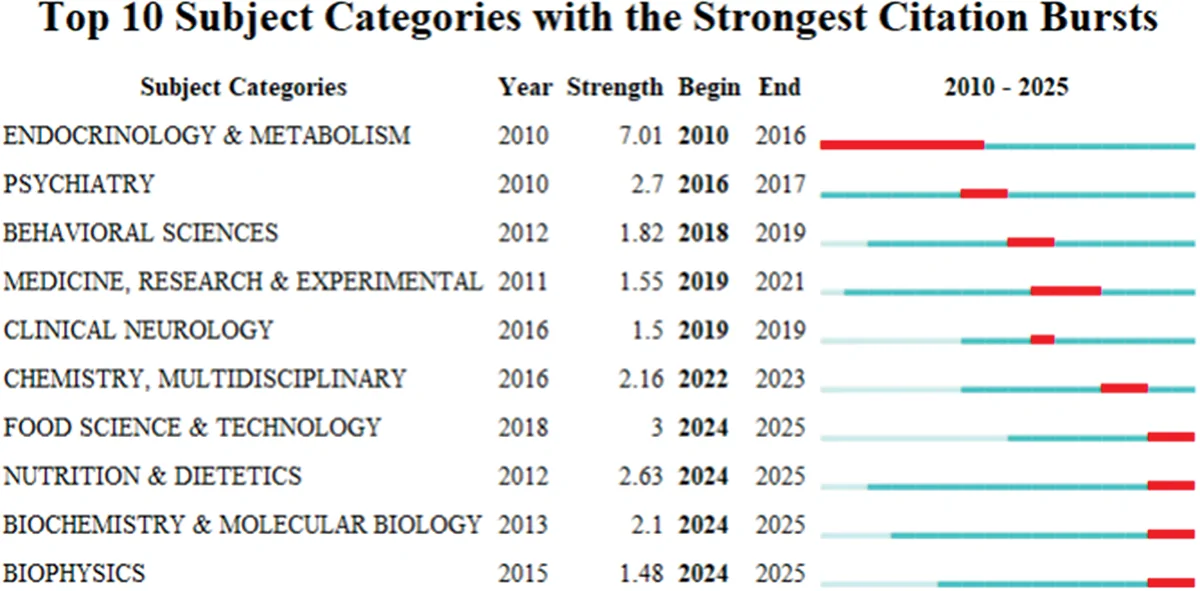
Top 10 subject categories with the strongest citation bursts in the research on neurobiological mechanisms of obesity and selected psychiatric conditions.

### Keyword analysis

VOSviewer identified 1,035 keywords; 32 occurred at least 10 times (Figure 6(A)). Four clusters centered on anxiety, depression, gut microbiota, the gut–brain axis, high-fat diet, HPA-axis dysfunction, inflammation, neuroinflammation, obesity, and stress. These keywords covered metabolic, immune, neural, and psychiatric themes. Keyword density visualizations stratified by document type showed overlapping thematic coverage between articles and reviews (Supplementary Figure 1). Both subsets included keywords related to obesity, depression, neuroinflammation, inflammation, and the gut–brain axis.

**Figure 6.**
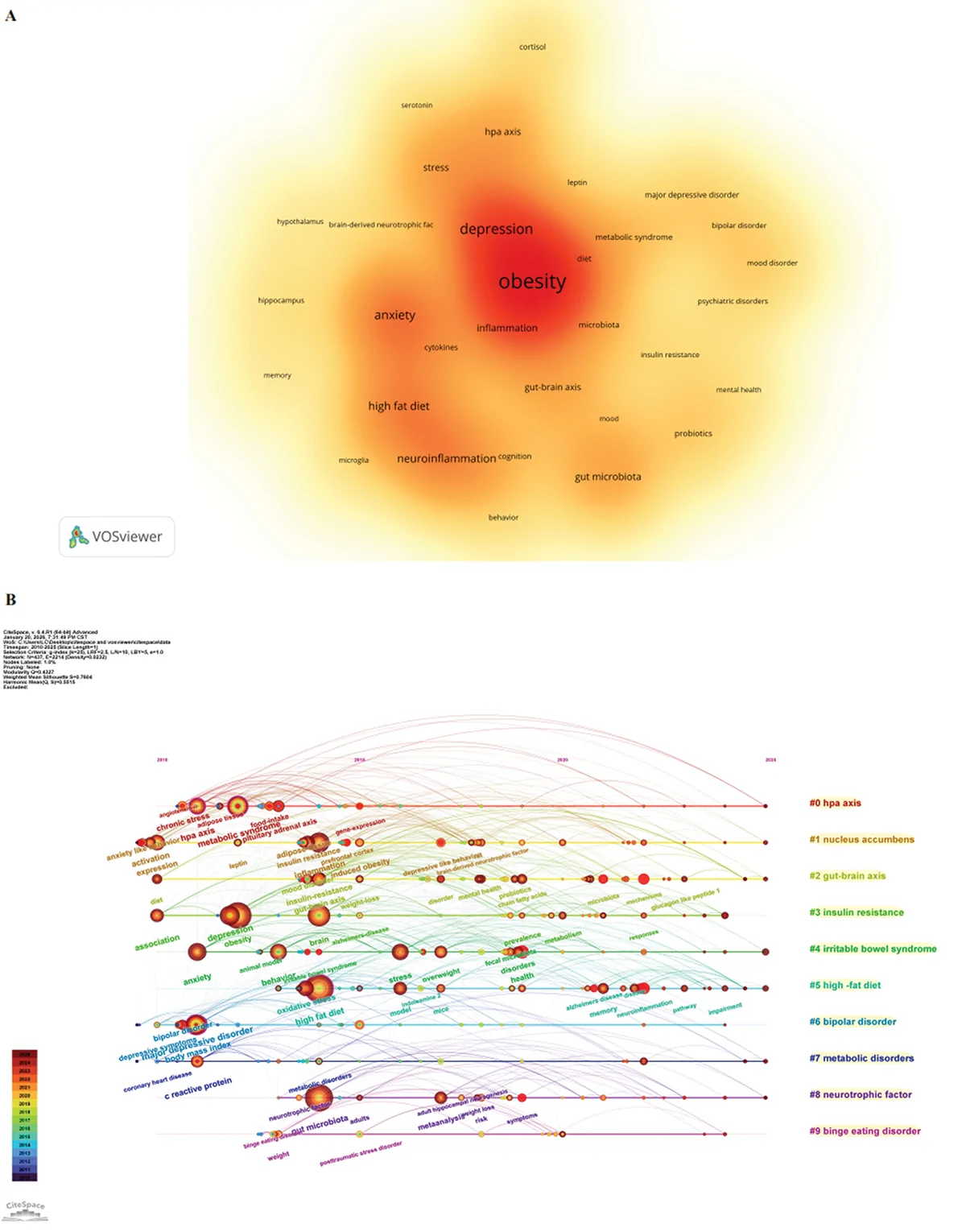
Visualization of keywords in the research field of neurobiological mechanisms of obesity and selected psychiatric conditions. A: Density mapping visualization of 32 keywords with a frequency of at least 10; B: Timeline visualization of keyword clusters.

The keyword timeline (Figure 6(B)) showed recent activity (2021–2025) in clusters including the HPA axis, nucleus accumbens, gut–brain axis, high-fat diet, metabolic disorders, and neurotrophic factors. Keyword burst analysis further showed temporal differences in the prominence of individual topics (Figure 7). Earlier burst terms included psychiatric phenotypes, stress-related processes, BMI, metabolic syndrome, and inflammatory markers, whereas terms appearing in intermediate periods included depressive-like behavior, gene expression, the prefrontal cortex, and central nervous system-related concepts. More recent burst terms included neuroinflammation, microbiota, mechanisms, and health. These patterns indicate changes in the temporal prominence of specific research topics within the analyzed literature, but do not necessarily imply that earlier topics declined, were replaced, or that the field followed a unidirectional progression.

**Figure 7.**
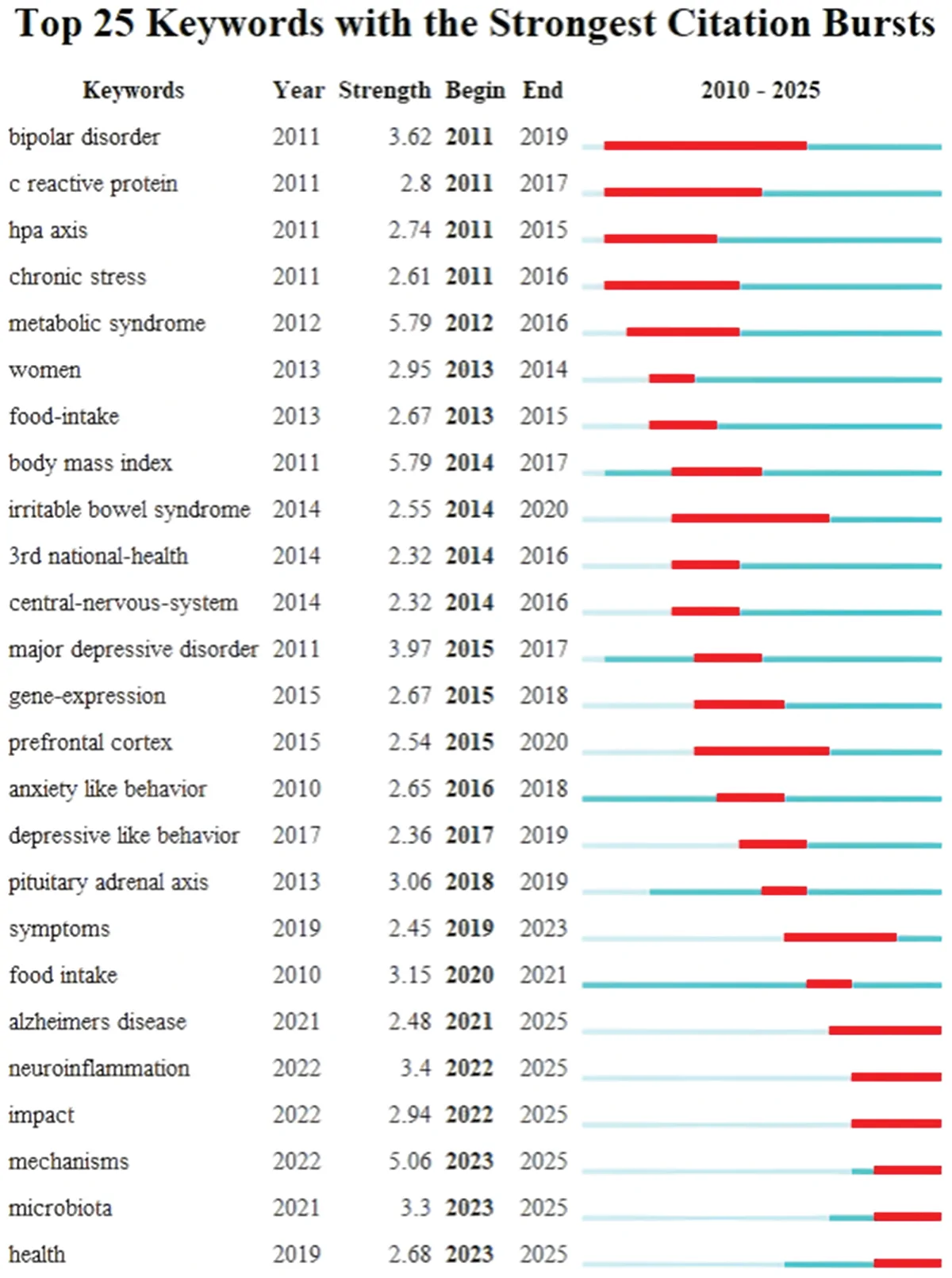
Top 25 keywords with the strongest burst strength in the research on neurobiological mechanisms of obesity and selected psychiatric conditions.

### Reference analysis

Reference co-citation analysis was performed using CiteSpace to characterize the intellectual structure of research on the neurobiological mechanisms of obesity and selected psychiatric conditions. Figure 8(A) presents the reference co-citation network generated by CiteSpace, and Table 4 summarizes the top 10 most frequently co-cited references ranked according to co-citation counts. Among these, the study by Fulton S (2022) investigated the overall threat of obesity to the prevalence of depression and anxiety and its potential public health impact [25]. Sharma S (2013) demonstrated that high-fat diet-induced obesity promotes the development of negative emotional states and depressive-like symptoms by elevating the levels of brain-derived neurotrophic factor (BDNF) and phosphorylated cAMP response element-binding protein (pCREB) within the striatum, specifically targeting the nucleus accumbens and dorsolateral striatum [26]. Dutheil S (2016) focused specifically on high-fat diet-induced anxiety and anhedonia, along with their effects on brain homeostasis and inflammation [27]. Meanwhile, André C (2014) demonstrated that diet-induced obesity can progressively impact cognitive, anxiety, and depressive-like behaviors through the activation of the cerebral indoleamine 2,3-dioxygenase pathway [28]. Guillemot-Legris O (2017) expanded the focus from the hypothalamus to whole-brain mechanisms of obesity-induced neuroinflammation [29]. Furthermore, the studies by Bruce-Keller AJ (2015) and Zheng P (2016) collectively revealed the independent role of obesity-related gut microbiota and metabolic remodeling in inducing neurobehavioural changes [30,34], while Kelly JR (2017) further confirmed through faecal microbiota transplantation experiments that depression-associated gut microbiota could directly induce neurobehavioural alterations [32]. Almeida-Suhett CP (2017) elucidated at a specific brain region level how a high-fat diet influences behavioural changes by upregulating IL-1β expression [31]. Finally, the review by Milaneschi Y (2019) integrated the common pathways of depression and obesity in terms of inflammation, metabolism, and neuroendocrinology from the perspective of shared biological mechanisms [33].

**Figure 8.**
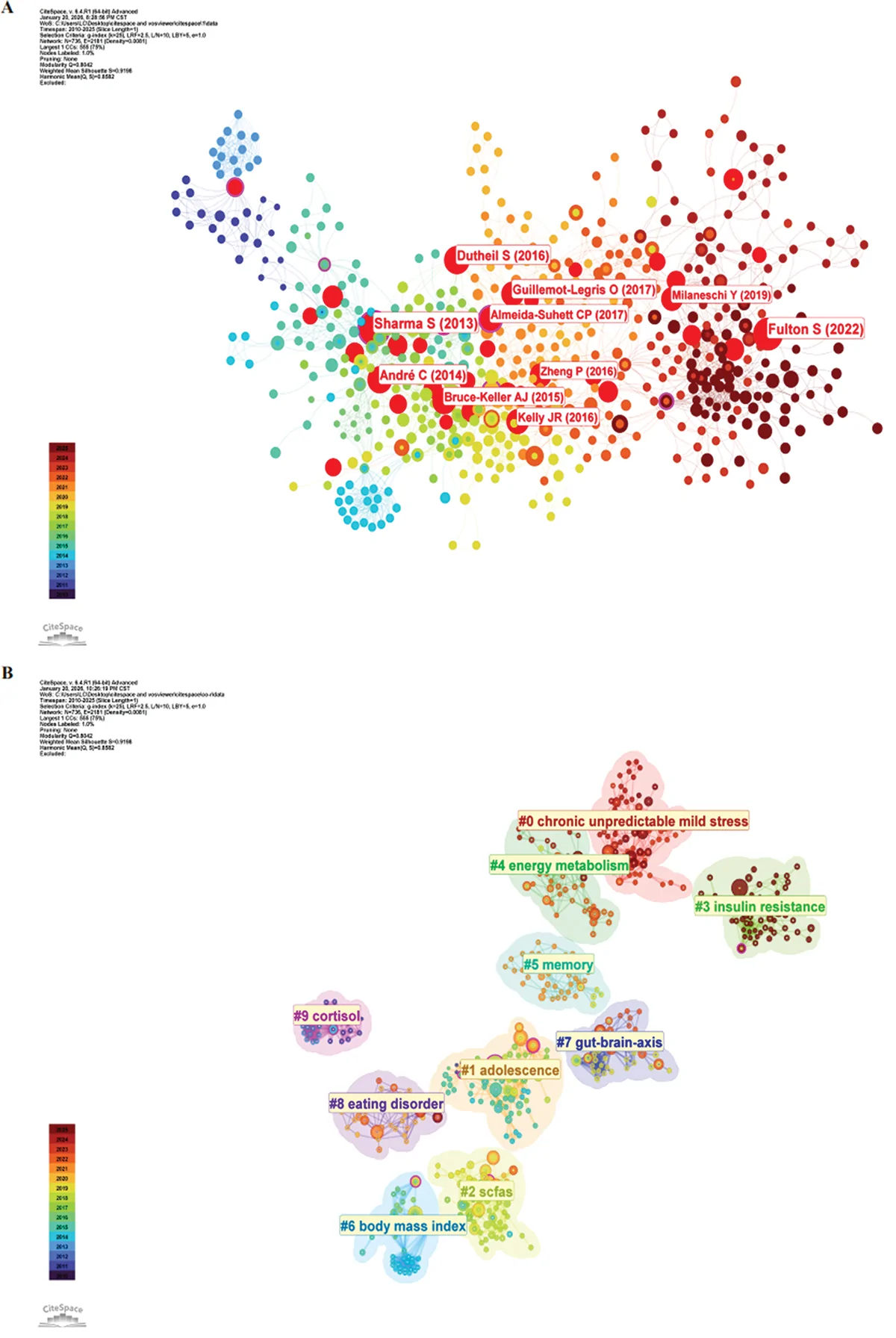
Visualization of co-cited references in the research field of neurobiological mechanisms of obesity and selected psychiatric conditions. A: Visualization of the reference co-citation network; B: Distribution of the top 10 major clusters of co-cited references.

**Table 4.** Top 10 most co-cited references.

Rank	Co-citation counts	Years	Node Name	First authors
1	26	2022	The menace of obesity to depression and anxiety prevalence	Fulton S [25]
2	25	2013	Diet-induced obesity promotes depressive-like behaviour that is associated with neural adaptations in brain reward circuitry	Sharma S [26]
3	20	2016	High-Fat Diet Induced Anxiety and Anhedonia: Impact on Brain Homeostasis and Inflammation	Dutheil S [27]
4	19	2014	Diet-induced obesity progressively alters cognition, anxiety-like behavior and lipopolysaccharide-induced depressive-like behavior: focus on brain indoleamine 2,3-dioxygenase activation	André C [28]
5	17	2017	Obesity-Induced Neuroinflammation: Beyond the Hypothalamus	Guillemot-Legris O [29]
6	16	2015	Obese-type gut microbiota induce neurobehavioral changes in the absence of obesity	Bruce-Keller AJ [30]
7	15	2017	Behavioral changes in male mice fed a high-fat diet are associated with IL-1β expression in specific brain regions	Almeida-Suhett CP [31]
8	15	2016	Transferring the blues: Depression-associated gut microbiota induces neurobehavioural changes in the rat	Kelly JR [32]
9	14	2019	Depression and obesity: evidence of shared biological mechanisms	Milaneschi Y [33]
10	14	2016	Gut microbiome remodeling induces depressive-like behaviors through a pathway mediated by the host’s metabolism	Zheng P [34]

CiteSpace identified 13 reference co-citation clusters, with the top 10 displayed in Figure 8(B). These clusters outline the thematic structure of research on the neurobiological links between obesity and selected psychiatric conditions. The ‘adolescence’ cluster (#1) highlights the developmental dimension of this literature, while ‘insulin resistance’ (#3) and ‘energy metabolism’ (#4) represent major metabolic themes. Additional clusters included ‘scfas’ (#2), ‘gut-brain-axis’ (#7), and ‘memory’ (#5), highlighting microbial metabolites, gut–brain communication, and memory-related research. The ‘eating disorder’ (#8) and ‘body mass index’ (#6) clusters further extend this thematic coverage to behavioral and anthropometric dimensions. Together, these clusters illustrate the breadth of developmental, metabolic, microbial, and behavioral topics represented in the co-cited literature. Citation burst analysis was performed using CiteSpace to identify references with rapid increases in citation activity during specific periods. Figure 9 presents the 25 references with the strongest citation bursts. The review by Fulton et al. (2022) had the highest burst strength (11.44) among the analyzed references. This review examines the biological mechanisms linking obesity with depression and anxiety through metabolic, inflammatory, and neurobiological pathways.

**Figure 9.**
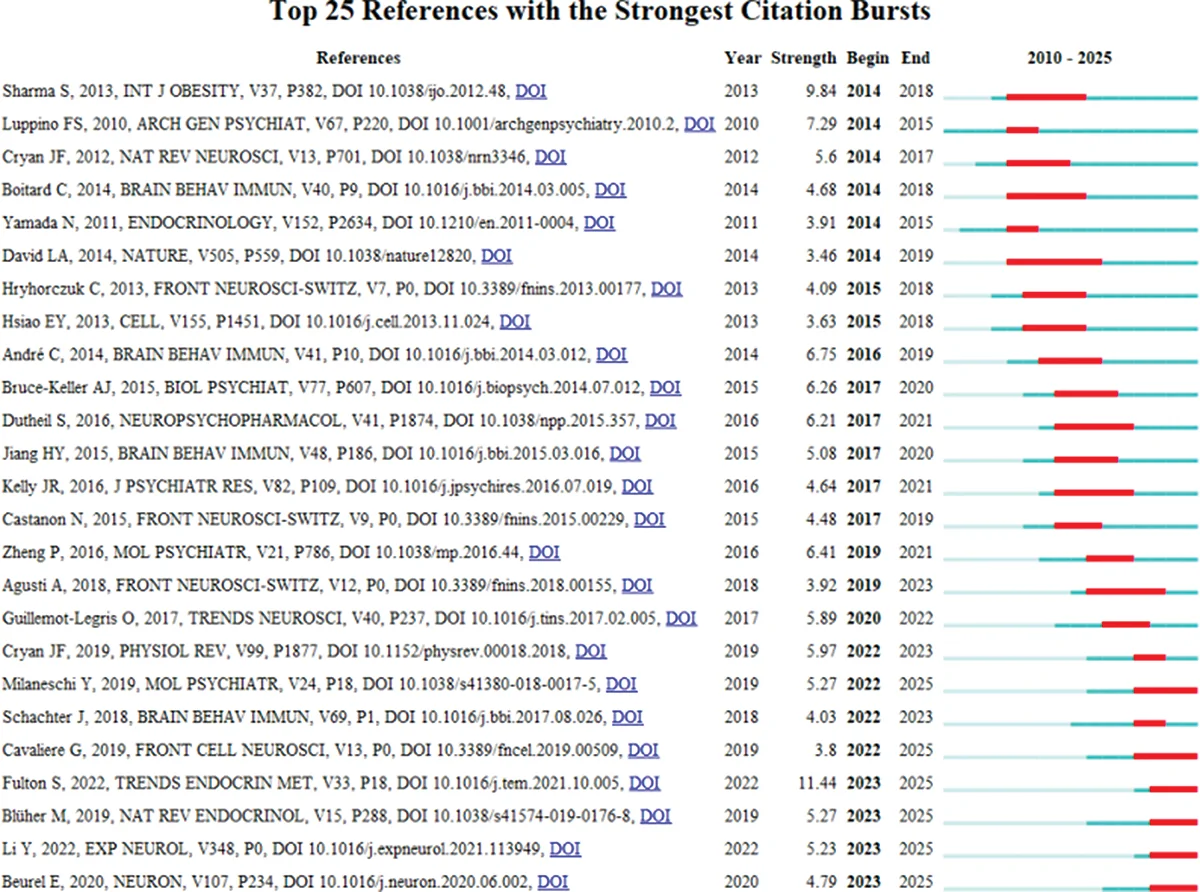
Top 25 references with the strongest citation bursts in the research field of neurobiological mechanisms of obesity and selected psychiatric conditions.

### Cross-database comparison

The final WoSCC and Scopus datasets contained 476 and 428 publications, respectively, with 362 shared publications. These represented 76.05% of the WoSCC dataset and 84.58% of the Scopus dataset. The union contained 542 distinct publications, yielding a publication-level Jaccard similarity coefficient of 0.668.

Annual publication counts were strongly positively correlated across 2010–2025 (Spearman’s ρ = 0.960; 16 paired annual observations; Figure 10(A)). Publication counts for the 10 countries displayed in Figure 10(B) also showed strong rank concordance (ρ = 0.969). The keyword sets displayed in Figure 10(C) each contained 32 terms, with 28 shared terms, yielding a Jaccard similarity coefficient of 0.778.

**Figure 10.**
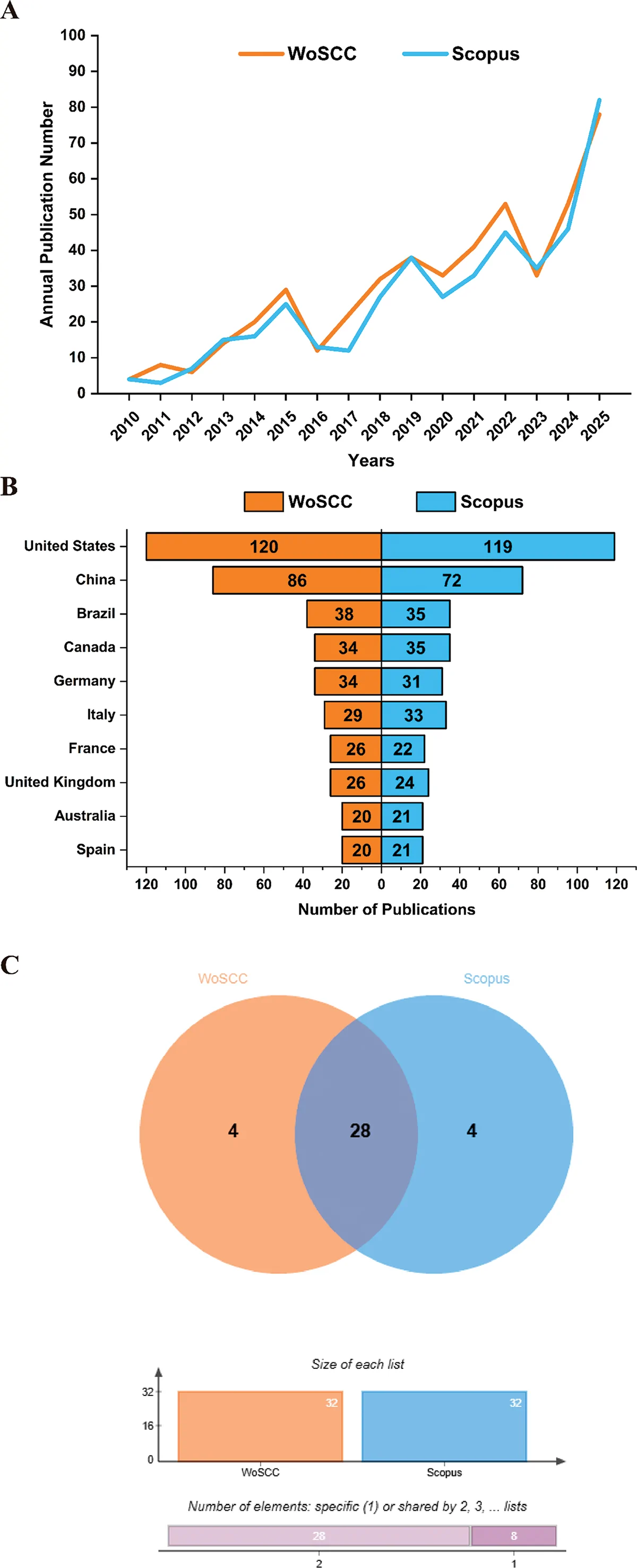
Cross-database comparison of selected bibliometric indicators between Web of Science core Collection and Scopus. A: Annual publication counts, 2010–2025 (Spearman’s ρ = 0.960); B: Publication counts for the 10 countries displayed (Spearman’s ρ = 0.969); C: Overlap between the selected high-frequency keyword sets (32 terms per database; 28 shared terms; Jaccard similarity = 0.778).

The Scopus keyword density map included themes also observed in WoSCC, including neuroinflammation, gut microbiota, the gut–brain axis, and high-fat diet (Supplementary Figure 2A). The corresponding keyword-cluster timeline is presented in Supplementary Figure 2B. Scopus burst analysis identified bursts for anxiety, diet, and cognitive function during 2023–2025, and for cafeteria diet during 2020–2025 (Supplementary Figure 3).

Together, these comparisons indicate agreement in selected aggregate patterns alongside differences in dataset composition. They provide a partial robustness assessment rather than evidence that all findings were unaffected by database choice.

## Discussion

### Summary of basic information: development trends of the field

Publications increased sharply after 2021, with the last 5 years accounting for 54.2% of the corpus. This growth parallels rising global burdens of obesity and mental disorders and the accumulation of epidemiological evidence for their bidirectional association [35–37].

The United States and China led publication output. Among the 10 most productive countries, France, Australia, and Canada had the highest mean annualized citations per publication. Although annualization accounts for differences in citation exposure time, these values are not field- and year-normalized and should not be interpreted as rankings of research quality or current influence. Eight of the ten most productive countries were high-income Western countries. Similar geographical concentration was reported in a bibliometric analysis of physical activity and cognitive function [38]. Although affiliations do not directly identify study populations, this pattern raises concerns about underrepresentation of populations in developing regions and highlights the need for geographically diverse research.

The author collaboration network showed several clusters, with relatively limited connections between clusters within the analyzed dataset. In the author co-citation analysis, Michael Maes had the highest co-citation count (96). These analyses describe complementary aspects of the literature: co-authorship reflects publication-based collaboration, whereas co-citation reflects authors being cited together within the included publications. Neither network establishes the stability of research teams, the extent of multicentre research, or the degree of conceptual or practical integration. The observed collaboration patterns raise questions for future research about opportunities for cross-group collaboration, but its potential benefits were not evaluated in this study.

### Knowledge structure and major research themes

Highly co-cited literature and reference clustering analysis collectively outline the major themes represented in the co-cited literature of the knowledge base in this field. Early foundational work (2013–2016), represented by Sharma S, André C, and Dutheil S, primarily focused on high-fat diet-induced depressive/anxiety-like behaviours and the underlying single-pathway mechanisms – either examining neuroadaptive changes in reward circuits [26], focusing on IDO activation-mediated tryptophan metabolic deviation [28], or exploring inflammatory responses in specific brain regions [27]. These studies provided crucial mechanistic clues for understanding the diet-emotion association.

Mid-term research (2015–2017), represented by studies by Bruce-Keller AJ, Zheng P, and Kelly JR, increasingly used microbiota-transfer experiments to investigate potential mechanistic links between gut microbial alterations and neurobehavioral outcomes [30,32,34]. These experimental studies provided evidence that transplantation of microbiota derived from obese or depressed donors could alter behavioral and metabolic phenotypes in recipient animals. Collectively, this work broadened the literature from predominantly central nervous system-focused mechanisms toward increasing consideration of gut–brain communication.

Recent integrative work (2019–2022) has begun to systematically consolidate previously dispersed mechanistic findings to construct multi-level pathological network models. Cryan JF’s review on the gut-brain axis systematically sorted out the role of bidirectional microbiota-gut-brain communication in mental disorders [39]; Milaneschi Y’s review focused on shared biological mechanisms between depression and obesity, particularly the intersection of inflammation and metabolism [33]; while Fulton et al. integrated dietary factors, peripheral inflammation, neuroimmunity, neural circuit plasticity, and endocrine regulation into a single framework, revealing a multi-cascade pathway from peripheral metabolic dysregulation to central mood disorders [25]. Building on these works, Michael Maes further proposed the NIMETOX framework, integrating multiple pathological modules – such as peripheral immune imbalance, central inflammation, lipid metabolism dysregulation, oxidative stress, and gut-brain axis dysregulation – into a unified theoretical system, providing a significant knowledge map for the field [40]. Although this framework was developed primarily in the context of MDD, related inflammatory, metabolic, and gut–brain processes have also been discussed in studies of anxiety and bipolar disorder [11,41], raising the possibility of broader transdiagnostic relevance that requires further empirical validation. Collectively, these highly cited publications illustrate increasing attention to frameworks that consider multiple biological processes together rather than in isolation. Taken together, these findings indicate that neuroinflammation, gut–brain communication, hypothalamic–pituitary–adrenal axis regulation, metabolic dysfunction, and reward-related neural processes have emerged as recurrent and increasingly interconnected themes in the literature. However, their co-occurrence within the bibliometric landscape should not be interpreted as evidence of a unified causal mechanism, and the functional relationships among these processes require further experimental and clinical validation.

The temporal distribution of burst keywords provides additional context for these thematic patterns. Earlier burst terms included HPA-axis-related and inflammatory indicators such as ‘HPA axis’ and ‘C-reactive protein’; intermediate-period terms included ‘depressive-like behavior’ and ‘gene expression’; and more recent burst terms included ‘neuroinflammation’, ‘microbiota’, and ‘mechanisms’. These differences indicate changes in the temporal prominence of research topics and an increasing recent visibility of studies spanning metabolic, immune, microbial, and neural domains.

### Research priorities and evidence gaps

Burst detection of subject categories and keyword emergence analysis are consistent with a shift in the research frontiers of this field. Subject categories showing sustained bursts in the recent period (2024–2025) include ‘FOOD SCIENCE & TECHNOLOGY’, ‘NUTRITION & DIETETICS’, and ‘BIOCHEMISTRY & MOLECULAR BIOLOGY’; these bursts overlap temporally with sustained high burst strength of keywords such as ‘neuroinflammation’, ‘microbiota’, and ‘health’. This pattern is consistent with growing publication activity at the interface of nutrition, metabolism, and psychiatry, a topic often referred to as nutritional psychiatry [42,43].

This pattern is associated with two shifts in how the co-occurring literature is organized. First, the discussion of diet has moved from broad ‘dietary patterns’ toward specific nutrients, food components, and their metabolites; for example, polyphenolic compounds, ω-3 polyunsaturated fatty acids, and dietary fibre are discussed in relation to gut microbiota composition and metabolites such as short-chain fatty acids [44–46]. Second, microbiomics, metabolomics, and epigenetics appear together in the recent literature, and several reviews propose a multi-step framework linking dietary exposure, gut microecology, metabolic remodeling, neuroimmunity, and behavioural change [47,48]. Together, these patterns are consistent with a move from single-pathway analyses toward more integrated, multi-level descriptions [49].

The burst-reference list points to two particularly influential consolidations. Milaneschi Y et al. (2019, Molecular Psychiatry) integrated shared biological pathways between depression and obesity across genetics, inflammation, and neuroendocrinology [33], and its citation burst persisted through 2025. Beurel E et al. (2020, Neuron) consolidated the bidirectional links between depression and inflammation, including the relevance of inflammatory cytokines and anti-inflammatory treatment responses to mood symptoms [50]. In the present corpus, the prominence of these two reviews reflects that inflammation is a recurring integrative theme rather than evidence that any specific pathway has been causally established.

Taken together, these patterns highlight gaps that future research in this area could address, rather than recommendations derived directly from the bibliometric data. Specific directions include: (1) large-scale longitudinal studies with multi-omics measurements to examine whether obesity–psychiatric subgroups can be reproducibly identified; (2) randomised studies of defined dietary components or patterns that measure both metabolic and mood-related outcomes; (3) controlled investigation of microbiota-targeted approaches (probiotics, prebiotics, postbiotics) in comorbid populations; and (4) neuroimaging studies that link dietary and microbial exposures to specific neural circuits. These directions are consistent with the recently burst keywords ‘neuroinflammation’, ‘microbiota’, and ‘health’, but their clinical and policy implications require direct empirical evaluation.

## Conclusion

This bibliometric analysis maps the evolving landscape of neurobiological research linking obesity with selected psychiatric conditions across 476 publications published between 2010 and 2025. Research output increased markedly after 2021, with neuroinflammation, the gut–brain axis, and hypothalamic–pituitary–adrenal (HPA) axis dysregulation emerging as recurrent themes. The literature also shows increasing attention to interactions among metabolic, immune, and neural processes rather than isolated biological pathways. Important gaps nevertheless remain. Causal and clinically oriented evidence is still limited, and many mechanistic insights are derived from high-fat diet-based animal models that may not capture the diversity of human dietary and nutritional environments. Future research should therefore prioritize causal validation across human and experimental models and assess the generalizability of proposed mechanisms across diverse populations and dietary contexts, particularly in developing regions. The hotspots identified here should be interpreted as priorities for further causal and translational investigation rather than as established mechanisms or evidence of intervention efficacy.

## Limitations

First, the primary analysis was restricted to English-language publications in the Web of Science Core Collection; differences in database coverage and indexing practices may have influenced the retrieved literature, although a supplementary Scopus analysis showed broad concordance with our main findings. The search also focused on selected psychiatric conditions rather than the full spectrum of mental disorders, and excluding Early Access records may have underrepresented the most recent literature. Second, citation-based indicators are influenced by publication age and database-specific practices, and we did not exclude self-citations, which may modestly inflate totals for highly productive authors and institutions. Publication bias may also shape the observable literature, although bibliometric analysis is not designed to detect it. In addition, keyword standardization required judgment calls, author and institutional labels were retained without manual disambiguation, and network inclusion thresholds and burst-detection parameters were selected for interpretability; different choices may yield somewhat different cluster structures. Third, despite predefined eligibility criteria, independent dual review, and inter-rater agreement evaluation, some subjectivity in screening was unavoidable. Finally, bibliometric analyses only depict publication and co-occurrence patterns and cannot establish causal or functional relationships. In addition, we did not perform further stratification of primary studies by research model (e.g. human clinical studies, animal experiments, or in vitro studies), which limits the differentiation of mechanistic and translational evidence levels. The identified emerging themes should therefore be regarded as priorities for further targeted experimental and clinical validation.

## Supplementary Material

Supplementary_Materials.docx

## Data Availability

Data will be made available on request.
