# Supplementary material for: Mapping the neurobiological landscape linking obesity with selected psychiatric conditions: global trends and emerging hotspots
Source: Glob Health Action. 2026 Oct 29;19(1):2749297. doi: 10.1080/16549716.2026.2749297 (PMC13721829; doi:10.1080/16549716.2026.2749297)
Supplement: Supplementary file 1 [file ZGHA_A_2749297_SM7681.docx]

Supplementary Material: Mapping the Neurobiological Landscape Linking Obesity with Selected Psychiatric Conditions: Global Trends and Emerging Hotspots

Supplementary Figures

Supplementary Figure 1. Keyword density visualizations stratified by document type. (A) Reviews; (B) Articles. For each subset, the 50 most frequent keywords occurring at least twice were visualized using VOSviewer. Warmer colors indicate higher local keyword density. Color intensity should not be directly compared between panels.

Supplementary Figure 2. Visualisation of keywords in the research field of neurobiological mechanisms of obesity and mental disorders based on the Scopus database (2010–2025). (A) Density visualisation of 32 high-frequency keywords. (B) Timeline visualisation of keyword clusters generated by CiteSpace.

Supplementary Figure 3. Citation burst analysis of keywords based on the Scopus database (2010–2025).

Supplementary Tables

Supplementary Table 1. Search strategies for Web of Science Core Collection and Scopus

Part A. Web of Science Core Collection

| Parameter | Detail |
| --- | --- |
| Database | Web of Science Core Collection |
| Database edition | Science Citation Index Expanded (SCI-EXPANDED) |
| Date of search | January 3, 2026 |
| Citation counts retrieved | January 3, 2026 |
| Time span（Publication Date） | January 1, 2010 – December 31, 2025 |
| Language | English |
| Document types included | Articles and Reviews |
| Document types excluded | Retracted publications, Conference Abstracts, Early Access, Editorials, Letters, Corrections, Book Chapters |
| Exact search string | TS=((obes* OR overweight OR “body mass index” OR BMI) AND (“mental disorder” OR “psychiatric disorder” OR “psychological distress” OR anxi* OR depress* OR “mood disorder” OR “bipolar disorder” OR “stress disorder” OR PTSD OR “post-traumatic stress disorder” OR “chronic stress”) AND (“neurobiolog” OR neurocircuit OR neuroinflamm* OR “HPA axis” OR “hypothalamic-pituitary-adrenal” OR “gut-brain axis” OR microbiota OR “neural pathway*”)) |

Part B. Scopus

| Parameter | Detail |
| --- | --- |
| Database | Scopus |
| Date of search | June 12, 2026 |
| Citation counts retrieved | June 12, 2026 |
| Time span（Publication Date） | January 1, 2010 – December 31, 2025 |
| Language | English |
| Document types included | Articles and Reviews |
| Exact search string | ( TITLE-ABS-KEY ( obes* OR overweight OR "body mass index" OR bmi ) AND TITLE-ABS-KEY ( "mental disorder" OR "psychiatric disorder" OR  "psychological distress" OR anxi* OR depress* OR "mood disorder" OR  "bipolar disorder" OR "stress disorder" OR ptsd OR "post-traumatic stress  disorder" OR "chronic stress" )  AND TITLE-ABS-KEY ( neurobiolog* OR neurocircuit OR neuroinflamm* OR  "hpa axis" OR "hypothalamic-pituitary-adrenal" OR "gut-brain axis" OR  microbiota OR "neural pathway*" ) )  AND PUBYEAR > 2009  AND PUBYEAR < 2026  AND ( LIMIT-TO ( LANGUAGE , "English" ) ) |

Supplementary Table 2. Study screening criteria, exclusion counts, and inter-reviewer agreement

Part A. Inter-reviewer agreement during study screening

Stage 1: Title and Abstract Screening (N = 1,677 records; observed agreement = 90.3%; Cohen’s κ = 0.79)

|  | Reviewer 2: Exclude | Reviewer 2: Retain | Row total |
| --- | --- | --- | --- |
| Reviewer 1: Exclude | 990 | 75 | 1065 |
| Reviewer 1: Retain | 88 | 524 | 612 |
| Column total | 1078 | 599 | 1,677 |

Stage 2: Full-Text Screening (N = 595 records; observed agreement = 94.8%; Cohen’s κ = 0.84)

|  | Reviewer 2: Exclude | Reviewer 2: Retain | Row total |
| --- | --- | --- | --- |
| Reviewer 1: Exclude | 102 | 15 | 117 |
| Reviewer 1: Retain | 16 | 462 | 478 |
| Column total | 118 | 477 | 595 |

Note: The contingency tables reflect the independent decisions of the two primary reviewers before consensus discussion. Final inclusion and exclusion decisions were determined after discussion between the two reviewers; unresolved cases were adjudicated by a third investigator. Cohen’s κ was used to quantify inter-reviewer agreement.

Part B. Screening criteria, exclusion counts, and representative eligibility examples

| Screening criterion | Operational definition | Title/abstract screening, n | Full-text screening, n | Total, n | Positive example (included) | Negative example (excluded) |
| --- | --- | --- | --- | --- | --- | --- |
| Core topic relevance | The study must substantively address both an obesity/overweight-related metabolic phenotype and a psychiatric condition or related phenotype captured by the predefined search strategy. Studies focused on another disease without a substantive obesity–psychiatric relationship were excluded. | 708 | 56 | 764 | A Systematic Review on the Association between Obesity and Mood Disorders and the Role of Gut Microbiota — directly examines obesity, mood disorders, and gut microbiota. | A systematic review of microbiome changes and impact of probiotic supplementation in children and adolescents with neuropsychiatric disorders — focuses on neuropsychiatric disorders and microbiome/probiotics without obesity as a substantive study component. |
| Neurobiological relevance | The study must examine a substantive biological or neural pathway potentially linking obesity/metabolic dysfunction with psychiatric outcomes, such as neuroinflammation, neuroendocrine regulation, neurotransmission, neural circuitry, adipokines, HPA-axis regulation, or gut–brain signaling. Studies limited to clinical/epidemiological association without neurobiological investigation were excluded. | 374 | 43 | 417 | Neuropsychiatric Abnormalities in Metabolic Disturbances: Interplay of Adipokines and Neurotransmission — examines adipokines, neurotransmission, HPA-axis dysregulation, and psychiatric abnormalities in metabolic disturbance. | The bidirectional association of nonalcoholic fatty liver disease with depression, bipolar disorder, and schizophrenia — examines disease association rather than a substantive neurobiological pathway linking obesity with psychiatric conditions. |
| Substantive thematic relevance | The obesity–psychiatric–neurobiological relationship must constitute a central or analytically substantive component of the study. Records in which these concepts appeared only incidentally, in background discussion, or without mechanistic integration were excluded. | - | 20 | 20 | The Role of Proopiomelanocortin and α-Melanocyte-Stimulating Hormone in the Metabolic Syndrome in Psychiatric Disorders: A Narrative Mini-Review — specifically evaluates POMC/α-MSH and neuroendocrine mechanisms connecting metabolic syndrome with psychiatric disorders. | Exploring the Relationship between Mood Disorders and Coexisting Health Conditions: The Focus on Nutraceuticals — the principal emphasis is broader comorbidity/nutraceutical management rather than a substantive neurobiological obesity–psychiatric mechanism. |
| Total |  | 1082 | 119 | 1,201 |  |  |

Supplementary Table 3. Geographical aggregation rules and keyword-standardization mappings

Part A. Geographical aggregation and name harmonization

| Original label | Standardized label | Processing rule |
| --- | --- | --- |
| taiwan | china | Geographical aggregation |
| england | united kingdom | Geographical aggregation |
| scotland | united kingdom | Geographical aggregation |
| wales | united kingdom | Geographical aggregation |
| turkiye | turkey | Country-name variant |
| bosnia & herceg | bosnia and herzegovina | Abbreviation expansion |

Part B. Keyword-standardization mappings

| Original label | Standardized label |
| --- | --- |
| hpa-axis | hpa axis |
| hypothalamic-pituitary-adrenal (hpa) axis | hpa axis |
| hypothalamic-pituitary-adrenal axis | hpa axis |
| hypothalamic-pituitary-adrenal stress axis | hpa axis |
| hypothalamic-pituitary-adrenal-axis (hpa-axis) | hpa axis |
| hpa | hpa axis |
| comorbidities | comorbidity |
| co-morbidity | comorbidity |
| binge-eating disorder | binge eating disorder |
| circadian rhythms | circadian rhythm |
| bipolar disorders | bipolar disorder |
| bdnf | brain-derived neurotrophic factor |
| c-reactive protein | crp |
| gut-brain-axis | gut-brain axis |
| brain-gut axis | gut-brain axis |
| the gut-brain axis | gut-brain axis |
| neurotrophins | neurotrophin |
| interleukin 6 | interleukin-6 |
| magnetic resonance imaging (mri) | mri |
| prebiotic | prebiotics |
| probiotic | probiotics |
| proopiomelanocortin (pomc) | pomc |
| rats | rat |
| diabetes mellitus 2 | type 2 diabetes mellitus |
| type 2 diabetes | type 2 diabetes mellitus |
| short -chain fatty acids | short-chain fatty acids |
| short chain fatty acid | short-chain fatty acids |
| psychiatric disorder | psychiatric disorders |
| neuro-inflammation | neuroinflammation |
| neuropsychiatric disease | neuropsychiatric disorders |
| neuropsychiatric diseases | neuropsychiatric disorders |
| memory impairments | memory impairment |
| high -fat diet | high fat diet |
| high fat diet. | high fat diet |
| high fatdiet | high fat diet |
| high-fat diet | high fat diet |
| high-fat diet (hfd) | high fat diet |
| high-carbohydrate diet | high carbohydrate diet |
| high-carbohydrate diet (hcd) | high carbohydrate diet |
| hfd | high fat diet |
| emotions | emotion |
| antidepressant | antidepressants |
| antidepressant (ad) | antidepressants |
| autism spectrum disorders | autism spectrum disorder |
| adolescents | adolescent |
| agouti-related protein | agrp |
| animal models | animal model |
| alcohol use disorder (aud) | alcohol use disorder |
| indoleamine 2-3-dioxygenase | indoleamine 2,3-dioxygenase |
| early-life stress | early life stress |
| children | child |
| antipsychotic agents | antipsychotics |
| diseases | disease |
| major depressive disorder (mdd) | major depressive disorder |
| major depressive disorders | major depressive disorder |
| mdd | major depressive disorder |
| mood disorders | mood disorder |
| neurobehaviors | neurobehavior |
| open-field | open field |
| sex differences | sex difference |
| ultraprocessed foods | ultra-processed foods |
| weigh loss | weight loss |
| bed | binge eating disorder |

Supplementary Table 4. Top 10 countries/regions by publication output without geographical aggregation.

| Rank | Countries | Publications | Citations | Average citations | Total link strength |
| --- | --- | --- | --- | --- | --- |
| 1 | United States | 120 | 9885 | 82.38 | 76 |
| 2 | China | 77 | 1871 | 24.30 | 16 |
| 3 | Brazil | 38 | 1728 | 45.47 | 30 |
| 4 | Canada | 34 | 2954 | 86.88 | 50 |
| 5 | Germany | 34 | 1314 | 38.65 | 34 |
| 6 | Italy | 29 | 724 | 24.97 | 33 |
| 7 | France | 26 | 2778 | 106.85 | 24 |
| 8 | England | 22 | 1440 | 65.45 | 38 |
| 9 | Australia | 20 | 1928 | 96.40 | 36 |
| 10 | Spain | 20 | 1385 | 69.25 | 38 |

Note: The sensitivity analysis shows that the top-10 country ranking is identical with and without geographical aggregation: the United States and China remain the two most prolific countries,France retains the highest average citation count (106.85), and no country enters or drops out of the top 10. Merging England, Scotland, and Wales into the United Kingdom increases its publication count from 22 (England alone) to 26 and its total link strength from 38 to 43, but does not alter its rank (8th) or the overall conclusions. Similarly, separating Taiwan from China reduces China’s publication count from 86 to 77 but does not change its rank (2nd). We therefore conclude that the main country-level findings are robust to the choice of geographical aggregation.

Supplementary Table 5. Top 10 journals by publication output on this topic

| Rank | Journals | Documents | Citations | Average citations |
| --- | --- | --- | --- | --- |
| 1 | Nutrients | 22 | 620 | 28.18 |
| 2 | Brain Behavior and Immunity | 17 | 760 | 44.71 |
| 3 | International Journal of Molecular Sciences | 15 | 346 | 23.07 |
| 4 | Psychoneuroendocrinology | 15 | 255 | 17.00 |
| 5 | Frontiers in Neuroscience | 10 | 257 | 25.70 |
| 6 | Scientific Reports | 7 | 434 | 62.00 |
| 7 | Molecular Neurobiology | 7 | 122 | 17.43 |
| 8 | Biological Psychiatry | 6 | 682 | 113.67 |
| 9 | Molecular Psychiatry | 6 | 570 | 95.00 |
| 10 | Neuroscience & Biobehavioral Reviews | 6 | 339 | 56.50 |

Supplementary Table 6. Top 10 journals by citation count on this topic

| Rank | Journals | Citations |
| --- | --- | --- |
| 1 | PLOS One | 789 |
| 2 | Brain Behavior and Immunity | 760 |
| 3 | Biological Psychiatry | 682 |
| 4 | Nature | 674 |
| 5 | Proceedings of The National Academy of Sciences of The United States of America | 648 |
| 6 | Psychoneuroendocrinology | 647 |
| 7 | Physiology & Behavior | 643 |
| 8 | Nutrients | 620 |
| 9 | Journal of Neuroscience | 572 |
| 10 | Molecular Psychiatry | 570 |
